# Exogenous cystine increases susceptibility of drug-resistant *Salmonella* to gentamicin by promoting oxidation of glutathione metabolism and imbalance of intracellular redox levels

**DOI:** 10.3389/fmicb.2025.1527480

**Published:** 2025-02-07

**Authors:** Junyuan Du, Zhiyi Wu, Chunyang Zhu, Heng Yang, Feike Zhao, Binghu Fang

**Affiliations:** ^1^Guangdong Provincial Key Laboratory of Veterinary Pharmaceutics Development and Safety Evaluation, South China Agricultural University, Guangzhou, China; ^2^National Risk Assessment Laboratory for Antimicrobial Resistance of Animal Original Bacteria, South China Agricultural University, Guangzhou, China

**Keywords:** *Salmonella*, gentamicin, cystine, resistance, metabonomics

## Abstract

**Introduction:**

Antibiotic overuse has caused the development of bacterial resistance, which is a major threat to public health. Intracellular metabolic processes are essential for maintaining the normal physiological activities of bacteria, and an increasing body of research has demonstrated a significant association between metabolic alterations and the development of drug resistance. Numerous studies have demonstrated that the addition of adjuvants can counteract bacterial antibiotic resistance.

**Method:**

Cystine treatment was verified *in vitro* to promote the lethal effect of gentamicin on *Salmonella* using *in vitro* bactericidal counting methods. The metabolic differences in *Salmonella enterica* Typhimurium standard strain ATCC 14028 with or without the addition of cystine were analyzed via untargeted metabolomics. The multifunctional electronic enzyme marker was used to determine intracellular reduced glutathione/oxidized glutathione (GSH/GSSG), ferrous iron on (Fe^2+^), and reactive oxygen species (ROS) levels. The expression of glutathione and stress genes was determined using real-time quantitative PCR.

**Result:**

We confirmed that exogenous cystine increased the lethal effect of gentamicin against strain *S. enterica* Typhimurium (ATCC 14028) and other clinically resistant *Salmonella* serotypes. Exogenous cystine stimulated the metabolism of the cell and activated the glutathione pathway while altering the GSH/GSSG ratio, which placed bacteria in a state of redox imbalance with increased Fe^2+^ and ROS levels. Our results suggest that when bacterial redox levels are reprogrammed, bacterial susceptibility to antibiotics can also change.

**Discussion:**

This study confirms that cystine enhances the antimicrobial efficacy of gentamicin against drug-resistant *Salmonella*. Through the application of metabolomics, the underlying metabolic mechanisms by which cystine exerts its effects on *Salmonella* have been elucidated, offering a novel perspective in the domain of metabolic reprogramming aimed at counteracting drug resistance. Furthermore, these findings reinforce the potential role of small-molecule metabolites as effective adjuvants to enhance antibiotic action.

## 1 Introduction

*Salmonella*, in the family Enterobacteriaceae, is a rod-shaped, gram-negative, flagellated, facultatively anaerobic bacterium (Feasey et al., 2012). It is a common zoonotic pathogen that is responsible for foodborne infections and was first identified by Eberth in the late 19th century (Hoelzer et al., 2011). The genus *Salmonella* contains two primary species, *Salmonella enterica* and *Salmonella bongori* (Cohen et al., 2022). The species *S. enterica* is further subdivided into more than 2,500 serotypes on the basis of surface antigens (Feasey et al., 2012).

Certain serotypes of *S. enterica* subspecies can induce acute or chronic gastroenteritis. For example, *S. enterica* Typhimurium infects a wide range of warm-blooded animals and is a significant contributor to the high global mortality associated with self-limiting diarrheal diseases (Hoelzer et al., 2011; Patra et al., 2021). Studies have shown that *Salmonella* causes approximately 2.8 billion cases of diarrhea annually, of which between 16 and 33 million are attributable to *S. enterica* Typhi, which result in an estimated 500,000–600,000 deaths. Additionally, infections caused by nontyphoidal *Salmonella* (NTS) account for 90 million cases, with approximately 155,000 fatalities (Bula-Rudas et al., 2015). *Salmonella enterica* Typhi is the causative agent of enteric fever (EF), which is rare in developed nations; most cases have been linked to international travel (Francois et al., 2020). In contrast, in underdeveloped countries with poor public health infrastructure, the incidence of EF is estimated to be approximately 100 cases per 100,000 people annually (Kim et al., 2022; Pitzer et al., 2019).

For developing countries with inadequate sanitation, the impact of NTS infections is particularly evident, and even developed countries with advanced sanitation and hygiene have a relatively high incidence of infection (Bula-Rudas et al., 2015). According to estimates by the Centers for Disease Control and Prevention (CDC), approximately 1.35 million cases of NTS infection occur annually in the United States, which result in 26,500 hospitalizations, 420 deaths, and direct health care costs estimated at $400 million (Kuehn, 2019). In 2014, *S. enterica* Enteritidis constituted 44.4% of the 16,000 locally reported infections in Germany, followed by *S. enterica* Typhimurium at 17.4% (2018, ECDC). In 2019, the European Union (EU) reported 87,923 cases of *Salmonella* infection, resulting in 16,628 hospitalizations and 140 fatalities (Weng et al., 2022). Furthermore, a study in 2020 revealed that *S. enterica* Typhimurium accounted for 40% of the isolates in Australia over the past two decades (Sodagari et al., 2020). Regions with lower economic development encounter disproportionately severe challenges associated with *Salmonella* infections. In Africa, 535,000 NTS cases were reported in 2017. Invasive nontyphoidal serotypes (iNTSs), which cause symptoms such as bacteremia and sepsis, are a significant concern (Uche et al., 2017). Approximately 85.8% of deaths related to iNTSs occur in sub-Saharan Africa, where HIV, immunodeficiency disorders (e.g., chronic granulomatous disease), malnutrition, and recent malaria or sickle cell anemia increase the risk of infection by iNTSs (Stanaway et al., 2019; Lazarus and Neu, 1975). In Thailand and the Lao People’s Democratic Republic, the prevalence of *Salmonella* in collected samples was 34.6 and 47.4%, respectively, with *S. enterica* Typhimurium detected at rates of 34 and 20.6%, respectively (Le Thi et al., 2017; Sinwat et al., 2016). On May 17, 2024, the World Health Organization (WHO) released the Bacterial Priority Pathogens List (BPPL2024), which identified 15 drug-resistant bacteria, including *Salmonella*, as a “high priority” (WHO, 2024). This shows that *Salmonella typhimurium* is a common and important serotype.

Currently, there is no vaccine for *Salmonella* (Baliban et al., 2020); thus, antibiotics remain crucial tools for treating infections such as *Salmonella*-induced gastroenteritis (Patra et al., 2021). Despite the availability of antibiotics, global morbidity and mortality caused by *Salmonella* remain substantial (Kirk et al., 2015), largely due to a increase in antibiotic resistance. Antibiotic resistance results from selective pressure caused by the use of antibiotics in microbial environments and is exacerbated by the overuse and inappropriate application of antibiotics (Prestinaci et al., 2015). Intensive farming practices, and their associated economic incentives, are a major contributing factor to bacterial antibiotic resistance, because antibiotics are used to treat or prevent bacterial diseases in food animals (Marshall and Levy, 2011). In addition, the emergence and dissemination of resistant bacterial strains are compounded by the practice of uncontrolled using antibiotics as growth promoters to increase poultry production (Oliver et al., 2011). Since 2006, antibiotic use for growth promotion has been prohibited in the European Union, and similar regulations have been enacted globally. Despite these measures, the escalation of antibiotic resistance and its associated challenges remain significant concerns.

Recent studies have demonstrated that altering bacterial metabolic states can restore the efficacy of antibiotics in drug-resistant strains (Zhao et al., 2021; Zheng et al., 2022). For example, the addition of exogenous pyruvate has been shown to increase the bactericidal activity of kanamycin against drug-resistant *E. coli* (Peng et al., 2015). Exogenous glutamate can enhance central carbon metabolism by promoting pyruvate cycling, which in turn increases the efficacy of aminoglycosides against *Escherichia coli* and *Edwardsiella tarda* (Su et al., 2018). Similarly, Leucine has been shown to increase the susceptibility of drug-resistant *Salmonella* to sarafloxacin by stimulating central carbon metabolism and activating electron transport chains, thereby increasing reactive oxygen species (ROS) production (Yang et al., 2023). These findings demonstrate that modifying bacterial biochemical metabolism through the addition of adjuvants can restore the sensitivity of drug-resistant bacteria to antibiotics.

Antimicrobial drugs include bactericidal and bacteriostatic drugs (Pankey and Sabath, 2004). The primary mechanisms of action of antimicrobial drugs have been extensively studied and can be categorized into three major groups: inhibition of cell wall synthesis, inhibition of protein synthesis, and inhibition of DNA replication and repair (Walsh, 2000). Notably, three classes of bactericidal antibiotics—β-lactams, quinolones, and aminoglycosides—have been reported to induce the production of harmful hydroxyl radicals in gram-positive and gram-negative bacteria, a process that activates the Fenton reaction (Kohanski et al., 2007; Belenky et al., 2015; Dwyer et al., 2014; Kumar A. et al., 2023; Kumar V. et al., 2023). The generation of hydroxyl radicals and the subsequent Fenton reaction are pivotal to the bactericidal efficacy of these antibiotics (Kohanski et al., 2007). The Fenton reaction, which requires hydrogen peroxide (H_2_O_2_) and ferrous iron (Fe^2+^), generates ROS. Cells produce ROS during normal aerobic respiration, and superoxide dismutase (SOD) in the cells catalyzes the conversion of superoxide anions (O_2_^−^) into hydrogen peroxide (H_2_O_2_) and oxygen (O_2_), thereby detoxifying ROS. However, when bacteria are exposed to excessive oxidative stress, which overwhelms their capacity to process ROS, they may undergo self-destruction (Zhao and Drlica, 2014). Oxidative stress is either endogenous or exogenous (Dryden, 2018). Endogenous oxidative stress arises from sources such as antibiotic treatment, cellular respiration, and intracellular redox reactions (Kim et al., 2019). Research has demonstrated that inducing a redox imbalance in bacteria can increase antibiotic-mediated bacterial death. For example, (+)-Catechin has been shown to exacerbate quinolone-induced redox imbalance in *A. baumannii* by increasing ROS production and decreasing reduced glutathione, and in combination thereby facilitating ciprofloxacin and gemifloxacin killing of *A. baumannii* (Ibitoye and Ajiboye, 2019). Similarly, exogenous glycine alters the intracellular redox state by promoting glutathione oxidation, which increases oxidative stress and restores the bactericidal activity of serum against *Vibrio alginolyticus* and *E. coli* (Kou et al., 2022). Consequently, research on modifying the redox level of drug-resistant bacteria to restore their sensitivity to antibiotics holds considerable promise. In this study, we demonstrated that exogenous cystine enhanced the bactericidal effect of gentamicin against drug-resistant *Salmonella* by activating the glutathione pathway while decreasing GSH/GSSG, thereby disrupting cellular redox homeostasis, increasing ROS production, and activating the Fenton reaction.

## 2 Materials and methods

### 2.1 Chemicals

Gentamicin sulfate, thiourea, other antibiotics, and dithiothreitol (DTT) were procured from Shanghai Maclean Biochemical Technology Co., Ltd. 2, 2′-Bipyridyl was obtained from Beijing Solepol Science and Technology Co. Cystine and cysteine were obtained from Shanghai Aladdin Biochemical Technology Co., Ltd. (Shanghai, China). Mueller Hinton (MH) broth, tryptic soy agar (TSA), Luria–Bertani (LB) broth, MacConkey agar, and MH agar were obtained from Guangdong Huankai Microbial Technology Co., Ltd. (Guangdong, China). M9 minimal medium was supplied by Shanghai ELITE Biotech Co., and high-performance liquid chromatography (HPLC)-grade methanol and acetonitrile were purchased from the Shanghai branch of Thermo Fisher Scientific (Waltham, MA, United States).

### 2.2 Bacterial strains

*Escherichia coli* (ATCC 25922) was obtained from the American Type Culture Collection (Manassas, VA, United States). The reference strain *S. enterica* Typhimurium (ATCC 14028) was sourced from the China Center of Industrial Culture Collection (Beijing, China). Clinically resistant *Salmonella* strains (S.R. Tm-1, S.R. Ld-2, and S.R. Db-3) were isolated from pig farms in the Xiyue region of South China (Guangdong, China). These isolates exhibited resistance to most aminoglycosides (Table 1). The default bacterial culture method was incubation in LB medium at 37°C with shaking at 200 rpm in a constant-temperature incubator.

**Table 1:** MIC value of different antimicrobials against *Salmonella* (μg/mL).

	ATCC 14028	S.R. Tm-1	S.R. Ld-2	S.R. Db-3
Gentamicin	1	64	64	64
Amikacin	2	32	8	16
Tobramycin	2	128	32	64
Spectinomycin	64	512	256	128
Apramycin	2	256	128	8
Ciprofloxacin	78/1,000	0.5	0.5	1
Amoxicillin	1	2,048	1,024	512
Ceftiofur	0.5	1	0.5	0.5
Ampicillin	1	1,024	1,024	256
Cefalexin	4	8	4	4

### 2.3 Determination of the minimum inhibitory concentration

In accordance with the CLSI guidelines, the minimum inhibitory concentration (MIC) was determined using the broth microdilution method. The procedure was as follows: Gentamicin was accurately weighed using an electronic balance with a precision of ±0.1 mg and dissolved in MH broth to prepare a gentamicin stock solution with a final concentration of 5,120 μg/mL. This solution was then filtered through a 0.22 μm membrane and stored at 4°C for future use. A 100 μL of MH broth was added to each well of a 96-well plate, and diluted gentamicin was added to the first well of each row, achieving a starting concentration of 512 μg/mL. Subsequent wells contained drug concentrations that were serially diluted by half, from the first well to the last. Overnight bacterial cultures were added to each well, with a final *Salmonella* concentration of 10^5^ CFU/mL. Both negative and positive controls were included. The 96-well plate was incubated at 37°C for 18 h, and the MIC was recorded as the lowest concentration of gentamicin in which no visible bacterial growth occurred. Each MIC determination had three biological replicates in a single trial, and all tests were repeated in at least three independent experiments to ensure reliability.

### 2.4 Nontargeted metabolomic assays and analysis

*S*. Typhimurium ATCC 14028 was cultured overnight in LB medium. The culture was then centrifuged at 4°C and 6,000 rpm for 10 min, and the bacterial pellet was washed twice with phosphate-buffered saline (PBS). The bacterial suspension was adjusted to 1 × 10^6^ CFU/mL and resuspended in 30 mL of M9 minimal medium. Cystine was added to the experimental group, whereas the control group received no cystine. The cultures were incubated in a constant temperature incubator at 37°C. After incubation, the bacteria were again washed twice with PBS following low-temperature centrifugation. The collected bacterial pellet was immediately quenched in liquid nitrogen to halt metabolic processes. Subsequently, 300 μL of 80% methanol aqueous mixture was added to the bacterial pellet, which was then vortexed for 30 s and sonicated for 6 min. The mixture was subsequently centrifuged at 5,000 rpm and 4°C for 1 min. The supernatant was transferred to a new centrifuge tube and lyophilized to obtain a dry powder. The resulting powder was reconstituted with 10% methanol solution to a suitable volume and subjected to liquid chromatography–mass spectrometry (LC–MS) analysis.

Chromatographic separation was performed using a Vanquish ultra high performance liquid chromatography (UHPLC) system with a column temperature of 40°C, a flow rate of 0.2 mL/min, and the mobile phase consisting of A: 0.1% formic acid and B: methanol. The gradient elution program was as follows: 0–1.5 min, 98% A; 1.5–3 min, linear decrease in A from 98 to 15%; 3–10 min, linear decrease in A from 15 to 0%; 10–10.1 min, linear increase in A from 0 to 98%; and 10.1–11 min, 11–12 min, A maintained at 98%. During the analysis, the samples were kept in a 4°C autosampler. To mitigate the effects of instrument signal fluctuations, samples were analyzed in a random order, and quality control (QC) samples were included to assess system stability and data reliability.

Mass spectrometry was conducted using a Q Exactive^™^ HF/Q Exactive^™^ HF-X with a mass–charge (M/Z) range of 100–1,500. The electrospray ionization source conditions were as follows: voltage at 3.5 kV, sheath gas flow at 35 psi, auxiliary gas flow at 10 L/min, and ion transmission tube temperature at 350°C. Both positive and negative ion modes were employed, and MS/MS data were acquired in data-dependent acquisition mode.

### 2.5 Antibiotic survival assay

We evaluated the effect of cystine on the bactericidal activity of gentamicin against *S*. Typhimurium ATCC 14028 and clinically resistant *Salmonella* strains. Initially, the bacterial strains were revived in LB medium and cultured until they reached the exponential growth phase. The cultures were then centrifuged at 6,000 rpm for 10 min at 4°C, after which the bacterial pellets were washed twice with PBS. The bacteria were subsequently resuspended at a concentration of 1 × 10^6^ CFU/mL in M9 minimal medium. Gentamicin sulfate at a 1 × MIC was added to the M9 minimal medium, and the strains were exposed to conditions with and without the addition of 2.5 mM, 5 mM, and10 mM cystine for a period of 8 h. Bacterial survival was evaluated every 2 h by counting colony-forming units (CFUs). The percentage of survival was calculated as the ratio of CFUs from the treated samples to those from the control samples.

To assess the survival of bacteria detected by the reduction of oxidized glutathione (GSSG) to reduced glutathione (GSH) by the addition of DTT, as well as to scavenge ROS by the addition of thiourea and to chelate divalent iron ions by the addition of 2,2′-bipyridyl, the strains were treated using the same methodology described above: 10 mM DTT or 500 μM 2,2′-bipyridyl was added to the medium, the mixture was incubated at 37°C for 8 h, diluted evenly on soybean casein agar medium, incubated overnight, and the number of CFUs was counted.

### 2.6 GSH/GSSG determination

The ratio of GSSG to GSH was assessed via a GSH and GSSG detection kit (Beyotime, China). Briefly, *Salmonella* was cocultured with cystine at a concentration of 1 × 10^6^ CFU/mL for 8 h. Following incubation, 5 mL of the bacterial suspension was centrifuged at 4°C at 6000 rpm for 10 min. The supernatant was discarded, and the bacterial pellet was resuspended in PBS and washed twice. To extract glutathione, protein removal reagent was added to the bacterial pellet, which was then subjected to rapid freezing in liquid nitrogen and thawing in a 37°C water bath freeze–thaw cycles twice, and then leave for 5 min. The mixture was subsequently centrifuged at 4°C and 10,000 rmp for 10 min. A total of 10 μL of the supernatant was removed, a GSH scavenger was added, and the mixture was allowed to react for 1 h at room temperature to remove the GSH. Another 10 μL of supernatant was allowed to stand under the same conditions for the same period of time, and the treated samples were used for the determination of GSSG and total glutathione. The total GSH content is calculated as twice the GSSG concentration of the sample, without the removal of GSH. The samples were added to the working solution and incubated at room temperature for 5 min, followed by the addition of the configured NADPH reserve solution; detection was performed with a multifunctional electronic enzyme marker.

### 2.7 Determination of ferrous iron content

The intracellular ferrous iron (Fe^2+^) content was quantified using an Elabscience Cell Ferrous Colorimetric Test Cassette. Briefly, Bacteria at a concentration of 1 × 10^6^ CFU/mL was cocultured with cystine in M9 minimal medium for 8 h. Next, 30 mL of the bacterial suspension was centrifuged at 4°C and 6,000 rpm for 10 min. The supernatant was discarded, and the bacterial pellet was resuspended in PBS and washed twice. Subsequently, 200 μL of lysis buffer was added to the pellet, and the cells were disrupted on ice with an ultrasonic cell disruptor for 5 min. The lysate was then centrifuged at 12,000 rpm for 10 min. A 160 μL aliquot of the supernatant was collected for further analysis. To determine the Fe^2+^ content, 80 μL of the supernatant was mixed with 80 μL of the chromogenic solution and incubated at 37°C for 10 min, and detected by a multifunctional electronic enzyme marker.

### 2.8 Determination of ROS levels

The ROS were assessed via an ROS assay kit (Beyotime, China). Briefly, the fluorescent probe 2′, 7′-dichlorodihydrofluorescein diacetate (DCFH-DA) was diluted and loaded into the cells. After incubation in the dark for 20 min with gentle shaking to ensure effective probe uptake, the samples were incubated at 37°C. The samples were then centrifuged at 4°C and 6,000 rpm for 5 min. The supernatant was discarded, and the bacterial pellet was washed twice with PBS to remove any unincorporated probes. The washed bacteria were resuspended in M9 Minimal medium; the initial concentration was adjusted to 1 × 10^6^ CFU/mL, and cultured with or without gentamicin sulfate and cystine. The ROS levels were measured at 2 h using an excitation wavelength of 488 nm and an emission wavelength of 525 nm. The relative ROS level was calculated as the ratio of the fluorescence intensity between the experimental and control samples.

### 2.9 RNA isolation and RT–qPCR

Cystine was added to the bacterial culture and incubated for 8 h. Following incubation, the bacterial pellet was collected by centrifugation, washed, and preserved for subsequent experiments. Total bacterial RNA was isolated using an RNAiso Plus kit (Takara, Japan). The RNA concentration and purity were assessed via an ultraviolet spectrophotometer (Tianheng, Shanghai).

For quantitative reverse transcription PCR (RT–qPCR), 1,000 ng of total RNA was reverse transcribed into complementary DNA (cDNA) using a Hifair^®^ II 1st Strand cDNA Synthesis Kit (Yeasen, China) following the manufacturer’s instructions. The RT–qPCR mixture consisted of 5 μL of SYBR Green Master Mix (Yeasen, Shanghai, China), 1 μL of upstream primer, 1 μL of downstream primer (with a final concentration of 0.2 μM for each primer), and 2 μL of cDNA, with enzyme-free water added to a final volume of 10 μL. PCR was conducted in a thermal cycler with the following parameters: initial denaturation was at 95°C for 5 min and was followed by 40 cycles of denaturation at 95°C for 10 s and annealing and extension at 60°C for 30 s. A melting curve was generated by heating from 60°C to 95°C at a rate of 0.05°C/s. The 16S rRNA gene was used as the reference gene. At least three biological replicates were used for statistical analysis. The primers used are listed in Supplementary Table S1.

### 2.10 Statistical analysis

Statistical analyses were conducted using GraphPad Prism software (version 10). Normality test method using Shapiro–Wilk test. Initially, the data were assessed for normality and homoscedasticity. Data were considered to follow a normal distribution and to exhibit homoscedasticity unless otherwise indicated. Significant differences were determined using an unpaired t-test. For RT–qPCR data, statistical significance was assessed using two-way analysis of variance (ANOVA). In all cases, the significance level was set to * indicates *p* < 0.05, ** indicates *p* < 0.01, *** indicates *p* < 0.001, and **** indicates *p* < 0.0001.

## 3 Results

### 3.1 Antimicrobial susceptibility of *S.* Typhimurium ATCC 14028 and three clinically resistant strains

The MIC results for *S*. Typhimurium ATCC 14028 and three clinically resistant *Salmonella* strains tested against various antibiotics are summarized in Table 1. The clinical strains of *Salmonella* referenced above exhibit resistance to the majority of aminoglycosides and are multidrug-resistant bacteria.

### 3.2 Cystine enhances the susceptibility of drug-resistant *Salmonella* to gentamicin

**Figure 1:**
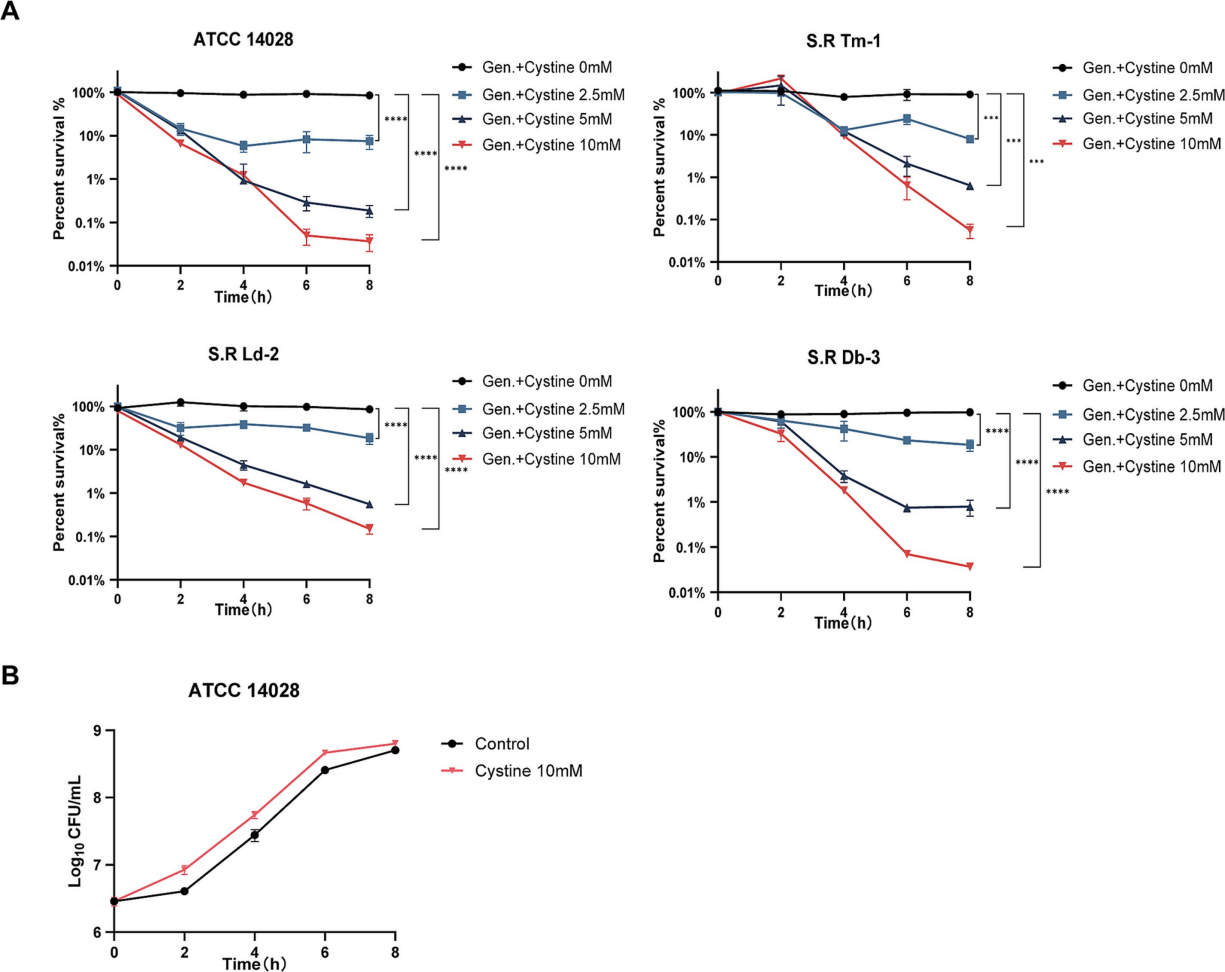
Killing curves for *S*. Typhimurium ATCC 14028 and three strains of clinically resistant *Salmonella*. **(A)** Survival curves for the test strains in M9 minimal medium supplemented with 1x MIC of gentamicin (Gen.) and cystine (0 mM, 2.5 mM, 5 mM, or 10 mM). **(B)** Bacterial growth curves for *S*. Typhimurium 14028 in M9 minimal medium with or without 10 mM cystine. The survival rate is the ratio of the calculated number of colonies in each experimental group to that in the blank control group. The results are displayed as the mean ± SEM, and three biological replicates were used. Differences were statistically significant according to Two-way ANOVA. An * indicates *p* < 0.05, ** indicates *p* < 0.01, *** indicates *p* < 0.001, and **** indicates *p* < 0.0001.

Exogenous cystine increased the susceptibility of drug-resistant *Salmonella* to gentamicin with a dose-dependent manner. To demonstrate this, *S*. Typhimurium ATCC 14028 and three drug-resistant *Salmonella* strains were incubated with 1x MIC gentamicin for 8 h in M9 medium with or without cystine at concentrations of 2.5 mM, 5 mM, and 10 mM. The number of viable cells was quantified over time, and the results are presented in Figure 1A. At each time point, the survival rate in the combination treatment group was significantly lower than that in the gentamicin-only group. For the four tested *Salmonella* strains, the use of 2.5 mM cystine in combination with gentamicin resulted in a 4.7- to 11.38-fold decrease in bacterial survival compared to gentamicin treatment alone. When the cystine concentration was increased to 10 mM, the reduction in bacterial survival ranged from 572- to 2,754-fold. We also found that the optimal dosage of cystine and the time to mortality were not consistent for different serotypes of *Salmonella*.

To rule out the possibility that cystine itself inhibited bacterial growth, 10 mM cystine was added to M9 medium, and bacterial growth curves were generated. The results demonstrated that cystine did not inhibit bacterial growth. Surprisingly, an increase in the growth rate was observed during the first 0–2 h (Figure 1B).

### 3.3 Cystine reprogramming of the *Salmonella* metabolome

**Figure 2:**
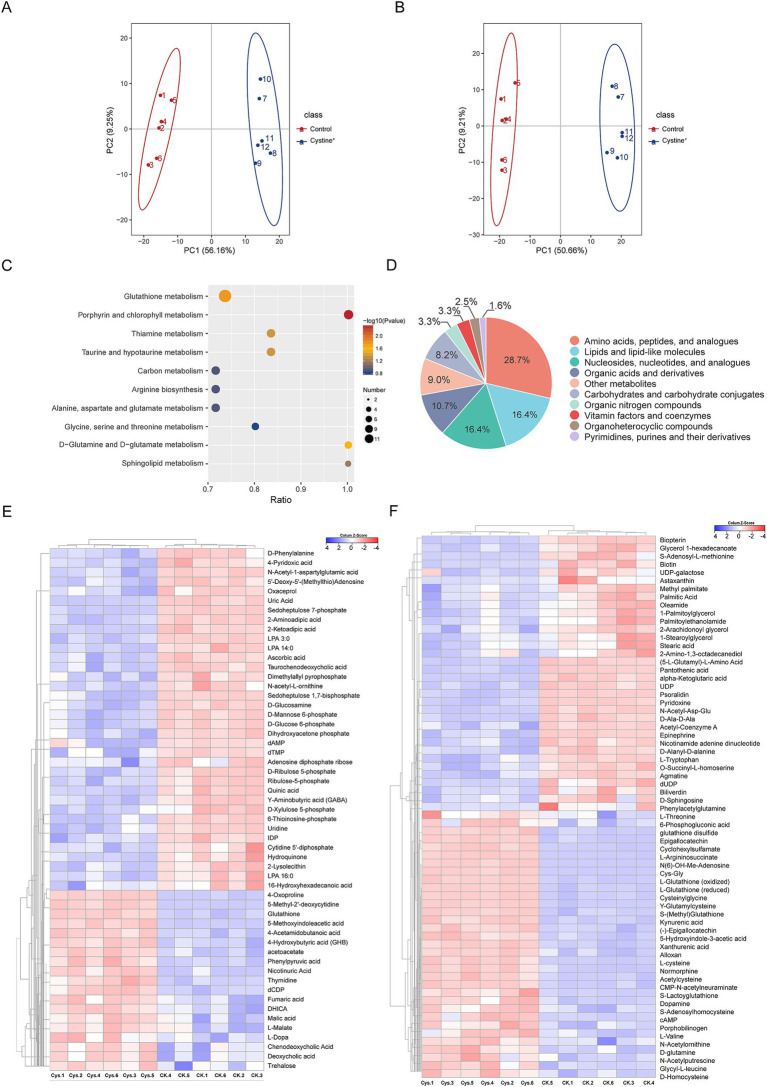
Metabolic profiling analysis. **(A)** PCA scores for *S*. Typhimurium ATCC 14028 cultured in cationic mode with and without added cystine. **(B)** PCA scores for *S*. Typhimurium ATCC 14028 incubated with and without added cystine in anionic mode. **(C)** Categorical pie charts of differentially abundant metabolites. **(D)** Metabolic heatmaps indicating increases and decreases in metabolites relative to median metabolite levels (see color scale). **(E)** Cluster analysis of different metabolites in anionic mode. “Ck” and “Cys” represent *S*. Typhimurium ATCC 14028 without and with cystine treatment, respectively. **(F)** Cluster analysis of different metabolites in cationic mode. “Ck” and “Cys” represent *S*. Typhimurium ATCC 14028 without and with cystine treatment, respectively.

To investigate the metabolic changes in *Salmonella* induced by exogenous cystine, we conducted a metabolomics reprogramming analysis of *S*. Typhimurium ATCC 14028 in the presence and absence of cystine. After 8 h of incubation, cell extracts were analyzed using UHPLC coupled with quadrupole time–flight mass spectrometry (UHPLC-Q-TOF MS), with six biological replicates per group. A total of 1,193 metabolites were identified, among which 507 metabolites exhibited significant alterations, including amino acids, lipids, carbohydrates, and nucleotides (Supplementary Table S2). To identify the metabolic differences, principal component analysis (PCA) was applied (Zhao et al., 2018). The PCA showed tight clustering of replicate samples and clear separation between the two groups (Figures 2A,B). The QC samples were tightly clustered, which confirmed a significant metabolic divergence between the cystine-treated and control groups.

The screening of differential metabolites was mainly referred to the three parameters of VIP, FC and *p*-value, VIP refers to the Variable Importance in the Projection of the first principal component of the PLS-DA model, and the VIP value indicates the contribution of the metabolite to the grouping; FC refers to the multiplicity of the differences (Fold Change), which is the ratio of the mean of all biological replicate quantification values of each metabolite in the comparison group; *p*-value was calculated by *T*-test, which indicates the significance level of difference. To identify significantly altered metabolites, orthogonal partial least squares-discriminant analysis (OPLS-DA) was employed, with VIP scores >1, fold change (FC) > 1.2 or FC < 0.833 and *p* values <0.05 as the selection criteria. We screened a total of 122 metabolites with significant differences (Supplementary Table S3), including 67 detected in the cationic mode and 55 in the anionic mode. The top three classes of metabolites with the highest relative abundances were amino acids and peptides (28.7%), lipids and lipid-like molecules (16.4%), and nucleosides and nucleotides (16.4%, Figure 2D). Hierarchical clustering analysis was used to visualize these differentially abundant metabolites. Among these metabolites, 52 were upregulated and 70 were downregulated in response to cystine treatment (Figures 2E,F).

Metabolic pathway analysis was performed via MetaboAnalyst 6.0, which identified 10 significantly enriched pathways, including glutathione metabolism, porphyrin metabolism, thiamine metabolism, central carbon metabolism, and several amino acid biosynthesis pathways (Figure 2C). In particular, glutathione metabolism was highly enriched, which suggests that exogenous cystine may provide substrate for the pathway and play a critical role in restoring gentamicin susceptibility in drug-resistant strains.

**Figure 3:**
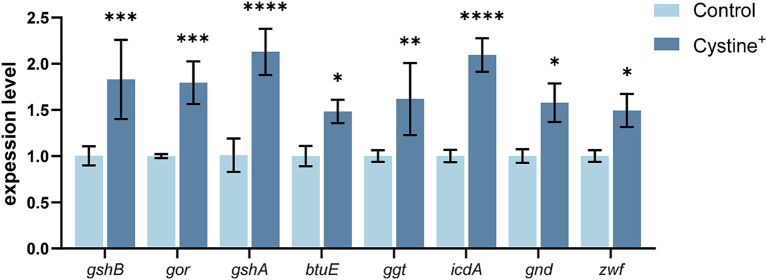
RT–qPCR for the expression of key glutathione genes in *S*. Typhimurium ATCC 14028 in the presence of 10 mM cysteine. Abbreviations: glutathione oxidoreductase (*gor*), glutathione synthetase (*gshB*), γ-glutamate-cysteine ligase (*gshA*), vitamin B12 transporter protein (*btuE*), γ-glutamyltransferase (*ggt*), isocitrate dehydrogenase (*icdA*), gluconate-6-phosphate dehydrogenase (*gnd*), and glucose-6-phosphate dehydrogenase (*zwf*). The results are shown as the mean ± SEM, and three biological replicates were used. Differences were statistically significant according to Two-way ANOVA. An * indicates *p* < 0.05, ** indicates *p* < 0.01, *** indicates *p* < 0.001, and **** indicates *p* < 0.0001.

To validate the hypothesis that exogenous cystine promotes the glutathione metabolic pathway, we quantified the expression of eight glutathione pathway-related genes via qPCR (Figure 3). The results demonstrated a significant upregulation in gene expression following 8 h of cystine treatment, indicating that cystine enhanced the glutathione metabolic pathway. The most pronounced changes were observed in the expression of γ-glutamate-cysteine ligase (*gshA*) and isocitrate dehydrogenase (icdA), which were upregulated by approximately 2-fold. Additionally, the expression of other related genes increased by approximately 50–70%. The associated metabolic processes included the synthesis of GSH and its conversion to GSSG. We then explored the relationship between glutathione biosynthesis and the enhanced mortality of drug-resistant *Salmonella* due to gentamicin in the presence of cystine.

### 3.4 Cystine disrupts cellular redox homeostasis by regulating GSH/GSSG

Glutathione, the most abundant intracellular thiol-containing compound, plays a critical role in cell defense against oxidative stress and various other cellular processes. The intracellular glutathione pool consists of GSSG (oxidized glutathione) and GSH (reduced glutathione). Numerous studies have shown that the GSH/GSSG ratio decreases when cells experience oxidative stress (Kou et al., 2022; Li et al., 2019; Wang et al., 2013). Therefore, the intracellular GSH/GSSG ratio is commonly used to assess oxidative homeostasis within the cell. We hypothesized that the addition of cystine would activate the glutathione pathway and alter the GSH/GSSG ratio, thereby disrupting bacterial redox homeostasis.

**Figure 4:**
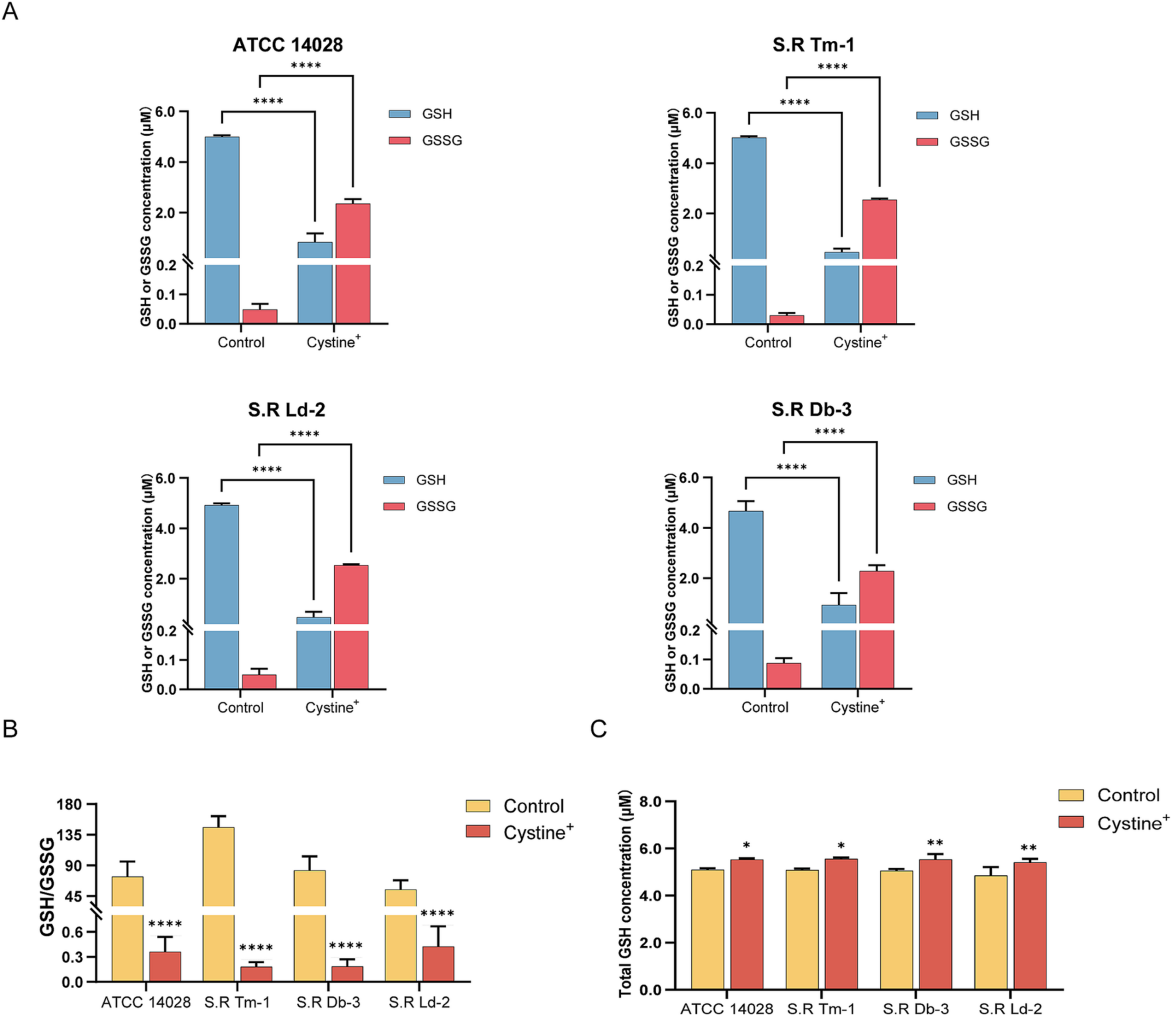
Determination of the intracellular GSH, GSSG, and total GSH contents and calculation of the GSH/GSSG ratio in *S*. Typhimurium ATCC 14028 and three clinically resistant *Salmonella* strains. **(A)** Concentrations of intracellular GSH and GSSG in experimental as well as control groups after addition of 10 mM cystine. **(B)** Intracellular GSH/GSSG ratio in the experimental as well as control groups in the presence of 10 mM cystine. **(C)** Total intracellular GSH in the presence of 10 mM cystine in experimental as well as control groups. The results are displayed as the mean ± SEM, and three biological replicates were used. Significant differences were identified via unpaired *t*-tests. An * indicates *p* < 0.05, ** indicates *p* < 0.01, *** indicates *p* < 0.001, and **** indicates *p* < 0.0001.

**Figure 5:**
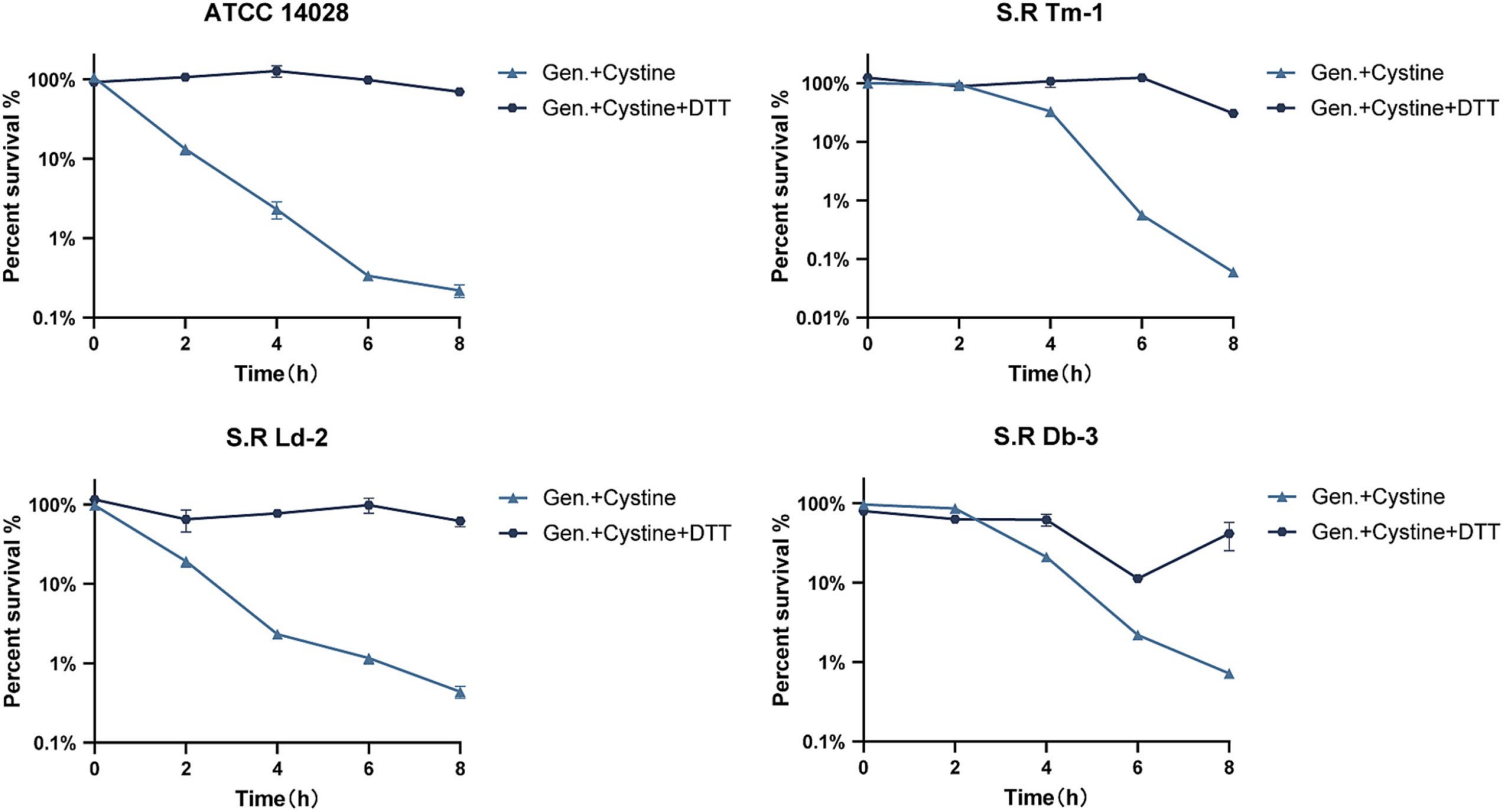
Survival curves for 1x MIC gentamicin, 10 mM cystine, and 10 mM DTT in M9 minimal medium for *S*. Typhimurium ATCC 14028 and clinically resistant *Salmonella*. The results are shown as the mean ± SEM, and three biological replicates were used.

To test this hypothesis, we added cystine to M9 minimal medium and measured the levels of total intracellular glutathione, GSH, and GSSG and the GSH/GSSG ratio after 8 h of incubation. The results indicated that for *S*. Typhimurium ATCC 14028 as well as three drug-resistant *Salmonella* strains, the total intracellular glutathione content increased by approximately 8–11% compared with that of the control group (Figure 4C). In the control group, intracellular GSSG accounted for approximately 1% of the total glutathione, which is consistent with previous findings that the normal intracellular GSH/GSSG ratio is approximately 100:1 (Dalle-Donne et al., 2008). However, in the presence of cystine, the intracellular GSSG levels of the tested strains increased significantly (nearly 45-fold), and the GSH/GSSG ratio decreased from 55–140 to 0.1–0.5 (Figures 4A,B). These data suggest that cystine disrupts intracellular redox homeostasis by elevating GSSG levels, which places cells in a state of oxidative stress. Furthermore, we found that the cystine-enhanced effect of gentamicin was eliminated by reducing GSSG to GSH with DTT treatment (Figure 5).

### 3.5 Cystine increases intracellular ferrous ion concentrations and elevates ROS levels following gentamicin treatment

Despite their specific drug–target interactions, bactericidal antibiotics are known to stimulate the production of highly deleterious hydroxyl radicals in bacteria, a process that is facilitated through the Fenton reaction involving intracellular ferrous iron (Kohanski et al., 2007). Our research confirms that cystine disrupts cellular redox levels by regulating GSH/GSSG, which in turn may enhance the Fenton reaction under antibiotic stress, thus creating favorable conditions for increased antibiotic-mediated bacterial death.

**Figure 6:**
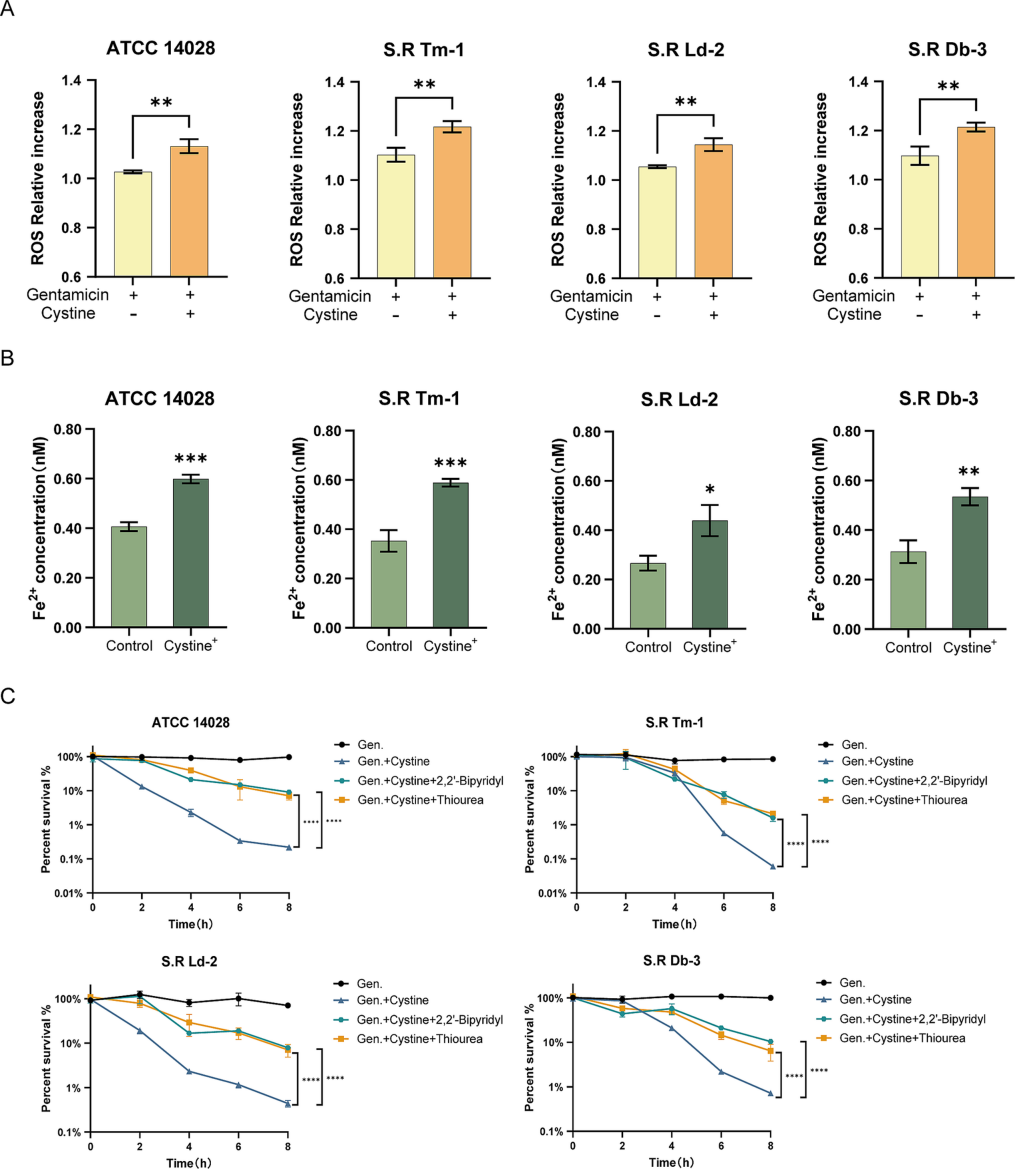
Cystine addition accelerates the Fenton reaction. **(A)** The ferrous ion concentration was elevated in the presence of 10 mM cystine. **(B)** In response to treatment with gentamicin, the addition of cystine resulted in elevated intracellular ROS concentrations. **(C)** Percent survival curves for 1x MIC, 10 mM cystine, and 150 mM thiourea or 500 μM 2,2′-bipyridyl in M9 minimal medium for *S*. Typhimurium ATCC 14028 and clinically resistant *Salmonella*. The results in **(A,B)** were analyzed by unpaired *t*-test, and the results in **(C)** were analyzed by two-way ANOVA, and the differences were statistically significant. An * indicates *p* < 0.05, ** indicates *p* < 0.01, and *** indicates *p* < 0.001.

Therefore, we measured ROS levels following gentamicin treatment in the presence or absence of cystine via a DCFH-DA fluorescent probe. Our results indicated that ROS levels were significantly greater in the cystine plus gentamicin group than in the gentamicin alone group (Figure 6A). When ROS were eliminated via the use of thiourea, a ROS scavenger, the enhanced bactericidal effect observed in the presence of cystine was markedly reduced (Figure 6C).

Next, we examined the levels of intracellular ferrous ions, which serve as substrates for the Fenton reaction. Compared with the control group, the addition of cystine significantly increased the concentration of intracellular ferrous ions (Figure 6B). Furthermore, the addition of the iron chelator 2, 2′-dipyridyl, which inhibited the Fenton reaction, resulted in a significant increase in bacterial survival in the cystine plus gentamicin group (Figure 6C). These findings demonstrate that the Fenton reaction plays a critical role in the synergistic bactericidal effects observed in the combined cystine and gentamicin treatment.

### 3.6 Cystine stimulates the expression of oxidative stress genes following gentamicin treatment

**Figure 7:**
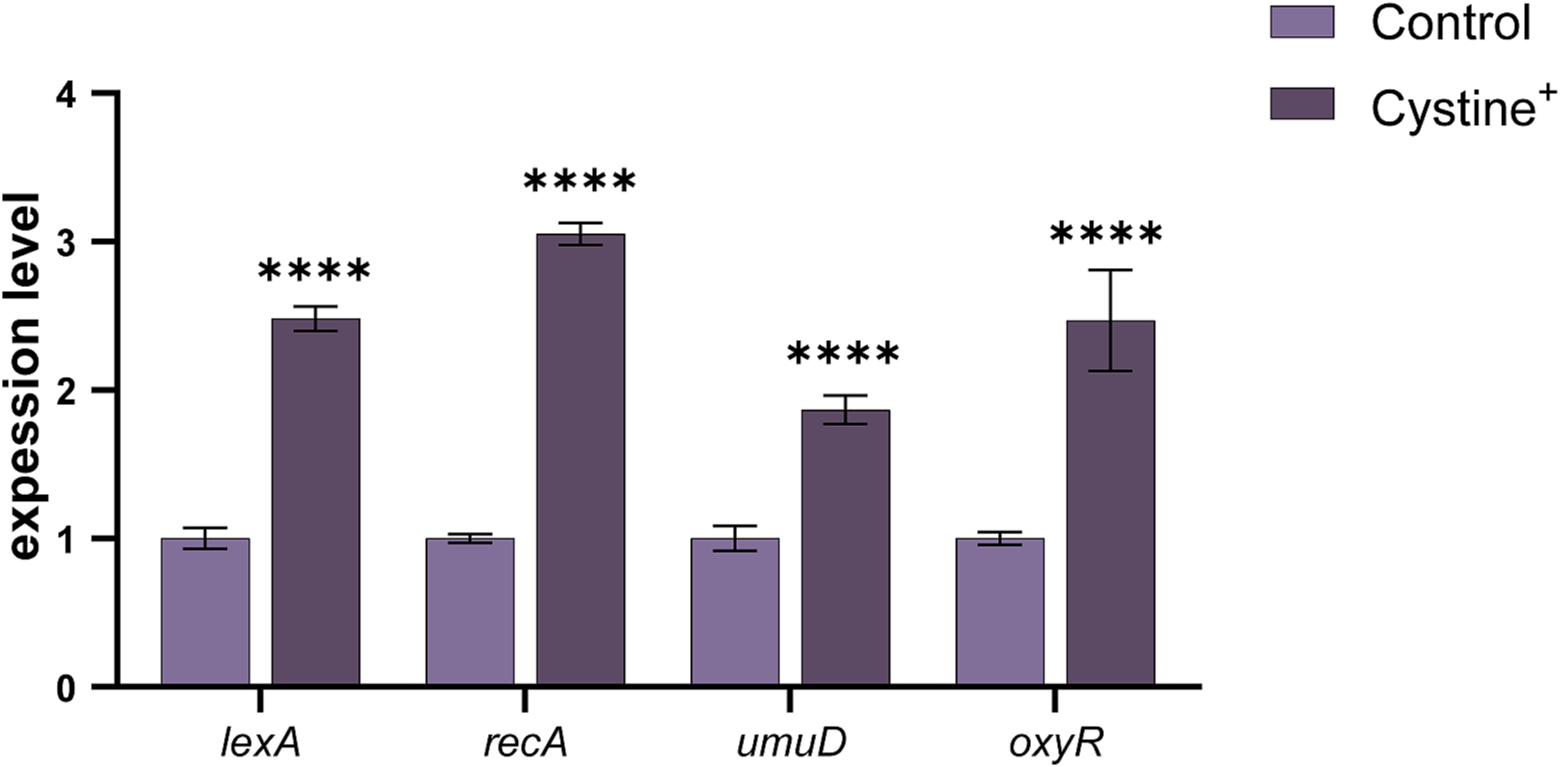
Cystine promotes oxidative stress gene expression. RT–qPCR analysis of stress and SOS gene expression in *S*. Typhimurium ATCC 14028 in the presence of 10 mM cystine. Abbreviations: *lexA*, SOS response regulator; *recA*, DNA strand exchange and recombination protein; *umuD*, error-prone repair: SOS-response transcriptional repressor; *oxyR*, regulatory protein sensor for oxidative stress. The results are displayed as the mean ± SEM, and three biological replicates were used. Differences were statistically significant according to Two-way ANOVA. An * indicates *p* < 0.05, ** indicates *p* < 0.01, *** indicates *p* < 0.001, and **** indicates *p* < 0.0001.

To further confirm that the addition of cystine induced an imbalance in intracellular redox levels, thereby causing cellular oxidative stress, we examined the expression of oxidative stress-related genes, including *OxyR*, and the SOS response genes *lexA*, *recA*, and *umuD* (Halsey et al., 2004; Kurasz et al., 2018). The results revealed significant upregulation in *lexA*, *recA*, *umuD*, and *OxyR* expression following the addition of cystine alone (Figure 7A), which aligned with our expectations. Among the genes related to SOS, *lexA, recA, and umuD* were up-regulated 1.8- to 3.1-fold, whereas the oxidative stress-related gene *OxyR* was up-regulated 2.5-fold. Interestingly, a previous analysis of *Salmonella* growth curves indicated that the upregulation of SOS and oxidative stress genes due to cystine addition did not significantly impact bacterial division and growth.

In summary, the addition of cystine resulted in the up-regulation of SOS and *OxyR* genes. This observation, along with the increased ROS levels, suggests that cystine enhances the bactericidal effect of gentamicin by exacerbating bacterial DNA damage and redox imbalance. These effects of cystine increase the susceptibility of bacteria to gentamicin.

## 4 Discussion

The rise of multidrug-resistant bacteria represents a significant threat to public health, and this threat is primarily driven by the overuse of clinical antibiotics. To mitigate bacterial resistance and enhance the efficacy of antibiotics, the use of antibiotic adjuvants is a promising strategy (Kumar A. et al., 2023; Kumar V. et al., 2023).

In this study, we evaluated the combined bactericidal efficacy of exogenous cystine in combination with gentamicin, screened cystine for synergistic antimicrobial effects, and selected three drug-resistant *Salmonella* serotypes for further analysis. During the cystine plus gentamicin assay, we observed that the optimal concentration of cystine needed to enhance the bactericidal action of gentamicin varied across strains. This variation may be attributed to the distinct biochemical characteristics of various *Salmonella* serotypes, including differences in cystine uptake and metabolism. Additionally, disparities in the response to oxidative stress among clinical strains adapted to diverse growth environments could also contribute to this variability. Additionally, we examined the effectiveness of other aminoglycosides (e.g., tobramycin, ampicillin, amikacin, and spectinomycin) across multiple resistant *Salmonella* serotypes. Interestingly, not all the strains experienced a synergistic effect. For example, tobramycin and spectinomycin exhibited synergy in certain strains, while ampicillin and amphotericin showed no synergy at all. Therefore, gentamicin, which had a better effect, was selected for subsequent studies.

We also investigated metabolic reprogramming, where the exogenous addition of specific compounds induces metabolic shifts in bacteria. Previous research highlighted similar phenomena; for example, glycine addition promoted metabolic transformations in *V. alginolyticus* (Kou et al., 2022), and glutamine restored antibiotic susceptibility in multidrug-resistant uropathogenic *E. coli* (Zhao et al., 2021). This approach is a potential strategy for reversing antibiotic resistance in drug-resistant bacteria. An increasing number of studies have thus focused on identifying metabolite-based adjuvants, including amino acids, nucleosides, lipids, and sugars. The central idea of metabolomic reprogramming involves leveraging changes in bacterial metabolism that potentially lead to the reversal or promotion of antibiotic resistance (Kou et al., 2022; Li et al., 2023; Ye et al., 2024).

Currently there are relatively few studies on the regulation of redox homeostasis through metabolic reprogramming, and more studies in this area have been conducted in terms of the TCA cycle, the P cycle, and the electron transport chain (Peng et al., 2015; Su et al., 2018; Tang et al., 2021). Therefore, our study provides a new insight into the field of metabolic reprogramming.

We conducted nontargeted metabolomics on the *S*. Typhimurium ATCC 14028 standard strain, with and without cystine treatment, using UHPLC-Q-TOF MS. Enrichment analysis of the differential metabolic pathways was performed for 122 different metabolites identified in both positive and negative ion modes. These metabolites were significantly enriched in the glutathione pathway. Notably, the upregulated metabolites were related primarily to glutathione metabolism, including key precursors for glutathione synthesis (*γ*-glutamylcysteine and acetylcysteine), metabolites involved in glutathione catabolism and its byproducts (cysteinylglycine and Cys-Gly), and both oxidized and reduced forms of glutathione (GSSG and GSH) and glutathione disulfide. Several upregulated metabolites, such as D-homocysteine and 4-oxoproline, are relatively understudied in bacteria, and their precise roles remain unclear. These compounds are recognized as biomarkers of oxidative stress in cardiovascular and neurological disorders (Gil et al., 2018; Stern et al., 2022; Tchantchou et al., 2021). In addition, increases in some methylated and oxo metabolites were also detected. Unexpectedly, the downregulated metabolites consisted predominantly of lipid classes. We speculate that it may be the oxidative stress caused by the addition of cystine that leads to an increase in ROS, which in turn destroys the lipids making them diffuse.

We subsequently investigated the expression of eight genes associated with the glutathione metabolic pathway: glutathione oxidoreductase (*gor*), glutathione synthetase (*gshB*), γ-glutamate-cysteine ligase (*gshA*), vitamin B12 transporter protein (*btuE*), γ-glutamyltransferase (*ggt*), isocitrate dehydrogenase (*icdA*), gluconate-6-phosphate dehydrogenase (*gnd*), and glucose-6-phosphate dehydrogenase (*zwf*). An overall upregulation of these genes occurred following cystine treatment, which indicated that cystine altered glutathione metabolism in bacterial cells. Based on these findings, we hypothesize that metabolic changes induced in the glutathione pathway contribute to the increased susceptibility of drug-resistant *Salmonella* to gentamicin treatment.

To identify the specific mechanisms by which cystine affects bacterial cells, we measured the GSH/GSSG ratios, intracellular ferrous iron concentrations, and ROS levels in bacteria cultured with exogenous cystine. The results demonstrated that cystine treatment caused a significant alteration in the intracellular GSH/GSSG ratio, with a marked increase in GSSG. Assessment of intracellular redox status can be accomplished by a variety of methods, each offering unique advantages. The Grx1-roGFP2 redox biosensor is capable of dynamic imaging and real-time quantification of intracellular GSH/GSSG in living cells with high sensitivity and accuracy (Gutscher et al., 2008). In addition, a GSH/GSSG assay kit was used in this study, which is capable of accurately quantifying GSH/GSSG in cells by utilizing the enzymatic reaction between 5, 5′-dithiobis-2-nitrobenzoic acid (DTNB) and glutathione reductase (GR). This method has been widely used for the determination of GSH/GSSG because of its simplicity, efficiency, and reliability. This method has been widely used for the determination of GSH/GSSG because of its simplicity, efficiency, and reliability (Hu et al., 2011). We found that this redox imbalance could be alleviated by the addition of DTT, which reduces GSSG to GSH, thereby eliminating the cystine-enhanced bactericidal effect of gentamicin. It has been previously suggested that the accumulation of GSSG may exert cytotoxic effects due to its propensity to form disulfides with intracellular proteins, which leads to their inactivation (Forman et al., 2009; Franco and Cidlowski, 2009).

We confirmed that the redox imbalance caused by exogenous cystine creates a cellular environment conducive to increased gentamicin -induced bacterial mortality. Similarly, phenolic acids were found to induce a redox imbalance and increase the mortality of drug-resistant *A. baumannii* by Polymyxin (Ajiboye et al., 2018), and exogenous glycine was shown to promote glutathione oxidation and restore bacterial susceptibility to serum-induced cell death (Kou et al., 2022). In our study, intracellular ROS levels and ferrous ion concentrations, which are key facilitators of the Fenton reaction, were significantly elevated following cystine treatment. We propose that cystine is transported into the bacterial cytosol and subsequently reduced to cysteine, a process reported to favor the generation of ferrous ions, which serve as electron donors for the Fenton reaction (Chonoles et al., 2015).

**Figure 8:**
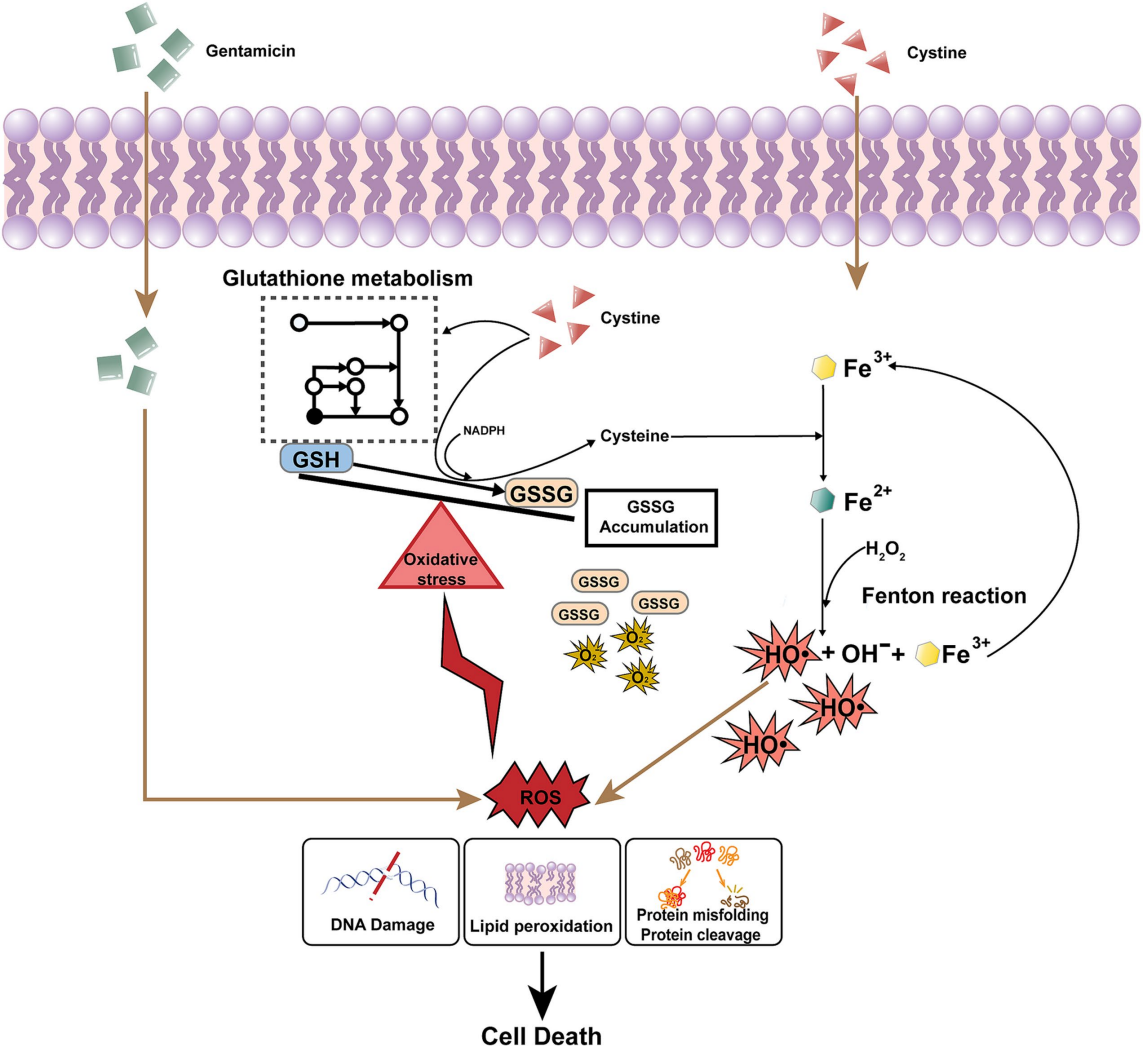
Mechanism underlying cystine and gentamicin-induced intracellular mortality in *Salmonella*. After cystine enters the cell, it promotes glutathione metabolism and the conversion of GSH to GSSG, which leads to the accumulation of GSSG and disruption of the intracellular redox balance. At the same time, it converts intracellular trivalent iron to divalent iron, and Fe^2+^ is involved in the Fenton reaction to generate hydroxyl radicals. Gentamicin permeates the bacterial cell membrane and induces the generation of ROS. ROS are highly cytotoxic, leading to significant cellular damage, including DNA strand breaks, lipid peroxidation, and the misfolding or cleavage of essential proteins. These oxidative effects compromise cellular integrity and function. Finally, cells are killed by lethal ROS levels due to the disruption of the redox balance induced by cystine and the Fenton reaction promoted by cystine.

Interestingly, the addition of cysteine alone did not enhance the bactericidal effect of gentamicin, which suggests that cystine has a specific role in this context. Previous research has indicated that cystine uptake may be uncontrolled in *E. coli*, with continuous transport into the cytosol leading to increased oxidative stress (Korshunov et al., 2020). Inside the cell, cystine is reduced to cysteine, which generates corresponding amounts of GSSG and further contributes to redox imbalance. This imbalance, caused by decreased GSH/GSSG ratios, places the cell in a state of oxidative stress, a phenomenon observed in both *E. coli* and *Salmonella* (Chonoles et al., 2015; Korshunov et al., 2020). Additionally, the excessive reduction of cystine to cysteine consumes large amounts of NADPH, a critical cofactor for maintaining cellular reducing power (Chandel, 2021) (Figure 8).

Finally, we confirmed that the use of iron chelators and ROS scavengers, such as thiourea, significantly reduced gentamicin-mediated mortality, which supports the hypothesis that the enhancement of the bactericidal action of gentamicin by cystine is mediated through the disruption of intracellular redox homeostasis. In conclusion, our findings suggest that cystine amplifies the bactericidal efficacy of gentamicin by disturbing the intracellular redox balance, thus sensitizing bacteria to oxidative stress and promoting cell death.

In this study, we demonstrated that cystine decreased the intracellular GSH/GSSG ratio in *Salmonella*, which disrupted cellular redox homeostasis, increased ROS production, and activated the Fenton reaction, thereby enhancing the bactericidal effect of gentamicin on drug-resistant bacterial strains. These findings provide a basis for the use of metabolomic reprogramming as a strategy to restore antibiotic susceptibility in resistant bacteria. Furthermore, these findings support the potential application of metabolites, such as cystine, as adjuvants to improve the efficacy of existing antibiotics, which opens a new avenue for addressing clinical antibiotic resistance.

## Data Availability

The data presented in the study are deposited in the figshare repository, accession number https://doi.org/10.6084/m9.figshare.28302719.v1.
