# Supplementary material for: Exogenous cystine increases susceptibility of drug-resistant Salmonella to gentamicin by promoting oxidation of glutathione metabolism and imbalance of intracellular redox levels
Source: Front Microbiol. 2025 Feb 7;16:1527480. doi: 10.3389/fmicb.2025.1527480 (PMC11843173; doi:10.3389/fmicb.2025.1527480)
Supplement: Supplementary file 1 [file Table_1.DOCX]

| **Primer** | **Sequence（**5'-3'**）** | **Product（**bp**）** |
| --- | --- | --- |
| gshB-F1 | GCGAAAGCGACTGGGAAATC |  |
| gshB-R1 | TTCAATTTCACGCACGCAGG | 140 |
| gor-F1 | CGACGAGCAGGTGAAAGTCT |  |
| gor-R1 | TTGGTAGCGCCCATCTTCAG | 195 |
| btuE-F1 | GAGCGGCTTTTACGAACGGA |  |
| btuE-R1 | CTGTACCACCTGACCATCCC | 112 |
| gshA-F1 | GGAAGGGCAGGACATAGAGC |  |
| gshA-R1 | TGCATCAAAGCGCCATAACG | 105 |
| ggt-F1 | CCGCTGAATAAAGTGGTGCG |  |
| ggt-R1 | TCACTTCGCTGCCGTAAGTT | 103 |
| icdA-F2 | AACAAATCCTGTTGCGTCCG |  |
| icdA-R2 | ATACCGATACCGCCAACCTG | 106 |
| gnd-F1 | AAACGCCTCGTCGTATCCTG |  |
| gnd-R1 | ATTGCGACGGATTGTGTCCT | 143 |
| zwf-F1 | CGGGGCTGGATCATAAGCAT |  |
| zwf-R1 | CCAGGCTTCTTCCACTTCGT | 167 |
| lexA-F1 | CAGACAGGTATGCCACCGAC |  |
| lexA-R1 | GACAAGCGGTAATCCGTCCT | 177 |
| recA-F1 | TCCGGTAAAACGACCCTGAC |  |
| recA-R1 | GTGCGTAAACAGGGTCAAGC | 106 |
| umuD-F1 | TGCTCGTTTCTCATCCCAGC |  |
| umuD-R1 | TCAGCGTTCCGTGAGCTATC | 115 |
| oxyR-F1 | AAGGTGCTCAAGGAGATGGC |  |
| oxyR-R1 | AGCTTCGGGAAAGTCTGGTG | 134 |
| 16S-F1 | CTGGAACTGAGACACGGTCC |  |
| 16S-R1 | GGAGTTAGCCGGTGCTTCTT | 199 |
